# Progeny Differentiation in Faba Bean Using Hyperspectral Images and Machine Learning

**DOI:** 10.1002/pld3.70193

**Published:** 2026-10-04

**Authors:** Rica‐Hanna Schlichtermann, Sebastian Warnemünde, Hanna Tietgen, Gregor Welna, Andreas Stahl, Benjamin Wittkop, Rod J. Snowdon

**Affiliations:** ^1^ Department of Plant Breeding Justus‐Liebig University Giessen Giessen Germany; ^2^ Julius Kuehn Institute (JKI), Federal Research Centre for Cultivated Plants Institute for Resistance Research and Stress Tolerance Quedlinburg Germany; ^3^ NPZ Innovation GmbH Holtsee Germany; ^4^ Norddeutsche Pflanzenzucht, Hans‐Georg Lembke KG Holtsee Germany

**Keywords:** breeding, heterosis, variety identification, xenia

## Abstract

Though currently a minor crop, faba bean is a promising source of plant‐based protein as global diets shift towards more plant‐based nutrition. To realize this potential, advances in breeding and cultivation are crucial. To exploit heterosis, faba bean breeding frequently utilizes synthetic cultivars, produced by open pollination of inbred lines, resulting in a mixture of F_1_ hybrid seeds and self‐pollinated offspring. Pure F_1_ hybrid cultivars are currently unavailable due to unstable cytoplasmic male sterility (CMS) systems. An ability to distinguish F_1_ seeds from their parental inbreds based on characteristics associated with the xenia effect could change this. The xenia effect refers to the influence of paternal pollen on seed traits, for example, seed weight and cotyledon cells in faba bean. In this study, we exploited the xenia effect captured in hyperspectral imaging data to develop machine learning scenarios for discriminating between parental and F_1_ seeds of open‐pollinated synthetic combinations (Syn‐1). The hyperspectral data were preprocessed using Savitzky–Golay filtering to reduce noise and smooth the spectra. Various machine learning algorithms were applied, incorporating Bayesian hyperparameter optimization. The scenarios achieved up to 98.9% accuracy in separating parental components of Syn‐1. When including all seeds, the model achieved an F_1_ score of 40.7%, indicating moderate detection and classification performance. As the harmonic mean of precision and recall, the F_1_ score reflects both the correctness of F_1_ seed identifications and the completeness with which F_1_ seeds were identified. While this approach does not yet enable the development of full hybrid cultivars, it paves the way for hybrid‐enriched cultivars. These could help to streamline breeding for synthetic cultivars and potentially increase yields, for example, by increasing the proportion of F_1_ hybrid seeds in synthetic cultivars. This study extends knowledge of the xenia effect in faba bean and provides a basis for further research aimed at enhancing breeding methods and productivity.

## 1 Introduction

With an increasing global population and a rising demand for plant‐based protein, production of faba bean ( *Vicia faba* L.) has expanded, underscoring its growing importance (Food and Agriculture Organization 2022; Sim et al. 2021). Faba bean offers significant benefits, such as a relatively strong ability to fix atmospheric nitrogen in symbiosis with *Rhizobium* bacteria, thereby providing residual nitrogen for subsequent crops (Aschi et al. 2017; Liu et al. 2019). This advantage is evident in improved yields of up to 20% in crop rotation systems that incorporate legumes compared to nonlegume systems (Zhao, Chen, et al. 2022). Additionally, faba bean seeds are characterized by a high protein and mineral content, making them suitable for use in alternative protein products, such as meat substitutes (Augustin and Cole 2022). Despite these benefits, faba bean suffers from yield instability, primarily due to drought and heat stress, necessitating further research, particularly in the breeding process, to fully harness its potential (Food and Agriculture Organization 2022; Bishop et al. 2016; Balko et al. 2023; Lavania et al. 2015).

Faba bean shows significant levels of heterosis (Stelling 1997; Link et al. 1996); although cytoplasmic male sterility (CMS) systems have been identified in faba bean (Bond et al. 1964; Palmer et al. 2011), these are not reliable, meaning that breeding of pure F_1_ hybrid cultivars is presently not possible. Instead, to at least partially exploit heterosis, faba bean is frequently bred as synthetic cultivars (Link 1990). This breeding process involves combinations of at least two selected inbred lines, which are propagated together in the field as a Syn‐0 combination. Open pollination and the allogamous nature of faba bean then facilitate outcrossing, resulting in a mixture of harvested seeds from F_1_ seed combinations between inbred lines along with self‐pollinated inbred seeds. Subsequent field propagation of this first synthetic generation (Syn‐1), again under open pollination, results in a Syn‐2 generation comprising a population of seeds from self‐pollinated parental genotypes, F_2_ progenies, parent × F_1_ crosses and (in cases where more than two founder parents were used) multicross hybrids (F_1_ × F_1_). Due to the heterozygous state, these synthetic generations tend to exhibit increased and more stable yields, showcasing heterosis. Highest yields are often achieved in Syn‐2 and Syn‐3 generations, depending on the number of parental components and their degree of self‐ and cross‐pollination; hence, up to two generations of propagation under open pollination may be performed (Stelling et al. 1994). This adds considerable time and expense to breeding operations. A considerably more efficient scenario to fully harness heterosis in faba bean, which can range from 33% to 51% for midparent yield heterosis (Link, Stelling, and Ebmeyer 1994), would be to breed F_1_ hybrids (Melchinger and Gumber 1998). However, in the absence of an effective CMS system, alternative strategies for exploitation of heterosis in the F_1_ generation might be advantageous to overcome the cost and effort associated with breeding of advanced‐generation synthetic cultivars.

In 1881, Focke described how fruits from crosses between *Raphanus sativus* and *Raphanus raphanistrum* could be distinguished from the two parents based on their color. He described the possible influence of different factors carried by pollen, indicating that hybridity could affect fruit characteristics. Focke (1881) termed this phenomenon “xenien.” Today, the term “xenien” or “xenia” is used to describe the general influence of pollen on fruit development, including color, shape, size, and chemical components (Denney 1992). Well‐known examples of traits showing a xenia effect include seed weight in maize and changes in color and shape in offspring of interspecific and intraspecific crosses in peas (Bulant and Gallais 1998; Sari et al. 2023). In faba bean, increases in seed weight and the number of cotyledon cells have been observed (Duc et al. 2001; Dieckmann and Link 2010). As proposed by Sari et al. (2023), xenia effects reflecting the influence of paternal pollen on the seed can be utilized to expedite crossing processes and reduce genotyping costs for validation of crosses.

Spectral data have been used for quality assessment in crop production and breeding since the 1960s, when near‐infrared reflectance (NIR) patterns were first shown to be associated with seed moisture content (Hart et al. 1962). More recently, it was demonstrated that hyperspectral images and machine learning could be used for seed variety identification in wheat, okra and loofah seeds, with accuracy ranging from 95% to 98% (Zhao, Que, et al. 2022; Nie et al. 2019). Extending the use of variation in seed NIR spectral patterns, Rincent et al. (2018) introduced the concept of phenomic selection. Roscher‐Ehrig et al. (2024) suggested that, especially at the start of a breeding programme, phenomic selection on the basis of NIR data could help to select superior genotypes, leading to better prediction accuracies at lower cost. New hyperspectral imaging technologies extend the wavelength range of reflectance measurements beyond the infrared and visible spectra. Here, we propose that linking hyperspectral imaging to the xenia effect in faba bean and other crops could enable a high‐throughput phenotyping approach that might facilitate the creation of F_1_ hybrid cultivars, or at least hybrid‐enriched synthetic cultivars. This can potentially help to implement the benefits of hybrid breeding, including improved yield and yield stability (Lee and Tollenaar 2007; Huang et al. 2017; Hackauf et al. 2022), into crops such as legumes for which establishment of traditional hybrid breeding systems based on biotechnological fertility control mechanisms has to date been unsuccessful.

This study investigates the use of hyperspectral imaging in combination with machine learning to differentiate parental components of biparental Syn‐1 seed combinations. The approach is further extended to distinguish F_1_ hybrid seeds from homozygous self‐pollinated seeds in Syn‐1 combinations, in order to analyze whether an observed xenia effect can be detected in this context. This could lead to potential applications in plant breeding to develop Syn‐1 cultivars enriched for a higher proportion of F_1_ hybrid seeds, either through accurate identification of F_1_ seeds or through classification and use of parental components that exhibit a higher outcrossing rate. In a more general context, it could also help breeders reduce costs for genotyping to confirm hybridity of offspring from controlled crosses.

## 2 Material and Methods

### 2.1 Plant Material

A total of 18 biparental, synthetic (Syn‐1), spring‐type faba bean combinations, along with pure‐breeding seeds from their respective parental lines, were obtained from NPZ Innovation GmbH (NPZi) (Holtsee, Germany) as part of the BreedPath project. Visual representation of the Syn‐1 combinations and the parental lines can be found in Figures S1 and S2. In this study, each Syn‐1 combination comprised seeds harvested from two parental components after open pollination of two more or less homozygous parental lines under isolation tents with bumblebees to guarantee outcrossing, meaning that the Syn‐1 comprises a mixture of F_1_ seeds from cross‐pollination and inbred seeds from self‐pollinations of the respective parental lines. Initially, 96 seeds from each Syn‐1 were analyzed along with 12 seeds from each parental line, and phenotypic data were collected for seed weight. Seeds were transferred to 24‐well tissue culture trays for hyperspectral imaging of each individual tray, as described in detail below. Individual seed images were extracted from the tray images using segmentation algorithms. Subsequently, the same procedure was performed using 300 additional seeds from two selected Syn‐1 combinations (NPZ‐BP3Y2023‐007 and NPZ‐BP3Y2023‐060, respectively). All spectral‐imaged seeds were subsequently grown, and DNA was extracted for marker analysis to determine whether each genotype corresponded to the F_1_ hybrid or one of the respective homozygous parents.

### 2.2 Overview of the Analysis Performed

The experiment was divided into two stages. Stage 1 focused on a global scenario including all 18 Syn‐1 combinations, with 96 seeds sampled from each Syn‐1 combination (scenario 1). First, using this global scenario, the parental components (the self‐pollinated plants) were classified, excluding the F_1_ seeds from the prediction. Furthermore, the classification capacity of the scenarios was assessed by investigating whether the F_1_ seeds could also be discriminated. To improve the prediction of F_1_ seeds in terms of F_1_ score and accuracy, different strategies were further applied to explore the full potential of this scenario. Previous studies showed that the xenia effect is more pronounced in more divergent genotypes (Duc et al. 2001). Hence, the Syn‐1 combinations were ranked according to the degree of spectral divergence between their respective parental components, in order to identify genotype‐specific differences. In Stage 2, Syn‐1 combinations with a high outcrossing rate and whose parental lines were categorized as belonging to more “distant” spectral groups were chosen for resampling with a higher number of seeds (300 more seeds per Syn‐1 combination, scenarios 2 and 3). Here, besides parental differentiation and simple F_1_ prediction, a wavelength‐specific approach was also applied, using only the spectral patterns where F_1_ seeds deviate from lying between the parental seeds. For scenario 3, the ability to distinguish F_1_ seeds using the spectral data was further investigated, separating parental components of the Syn‐1 based on a principal component analysis (PCA) (Table 1).

**TABLE 1:** Overview of used scenarios with target prediction, used data and applied strategies.

Stage	Scenario	Prediction	Data structure	Applied strategies
1	Scenario 1: 18 synthetic Syn‐1 combinations with 96 seeds each	Parental	Balanced	Simple
		F_1_	Resampling of crosses	Simple, Distance groups
2	Scenario 2 NPZ‐BP3Y2023‐060 with 300 additional seeds	Parental	Balanced	Simple
		F_1_	Balanced	Simple, specific wavelengths
	Scenario 3 NPZ‐BP3Y2023‐007 with 300 additional seeds	Parental	Balanced	Simple
		F_1_	Balanced	Simple, specific wavelengths, separated parental components

### 2.3 Hyperspectral Imaging

For hyperspectral imaging, seeds were placed in 24‐well tissue culture plates before sowing, with each seed being assigned a unique identifier. Hyperspectral imaging was conducted over a spectral range of 400–2500 nm. For this purpose, two hyperspectral line‐scanning sensors (Norsk Elektro Optikk A/S, Skedsmokorset, Norway) were used: (i) the HySpex VNIR‐1800, covering the visible and near‐infrared region (VNIR; 400–1000 nm) and (ii) the HySpex SWIR‐384, covering the short‐wave infrared region (SWIR; 1000–2500 nm). Image acquisition and radiometric calibration were performed using the camera vendor's software, HySpex Ground and HySpex Rad. In every image, a Zenith Polymer diffuser (Sphere Optics GmbH, Herrsching, Germany) was included for calibration. The camera frame period was adjusted by the image acquisition software to match the speed of the linear stage, thereby ensuring geometrically correct image acquisition. For illumination, a 1000 W short‐wave halogen spotlight (Hedler C12, Hedler Systemlicht, Runkel/Lahn, Germany) was installed. The setup is shown in Figure 1.

**FIGURE 1:**
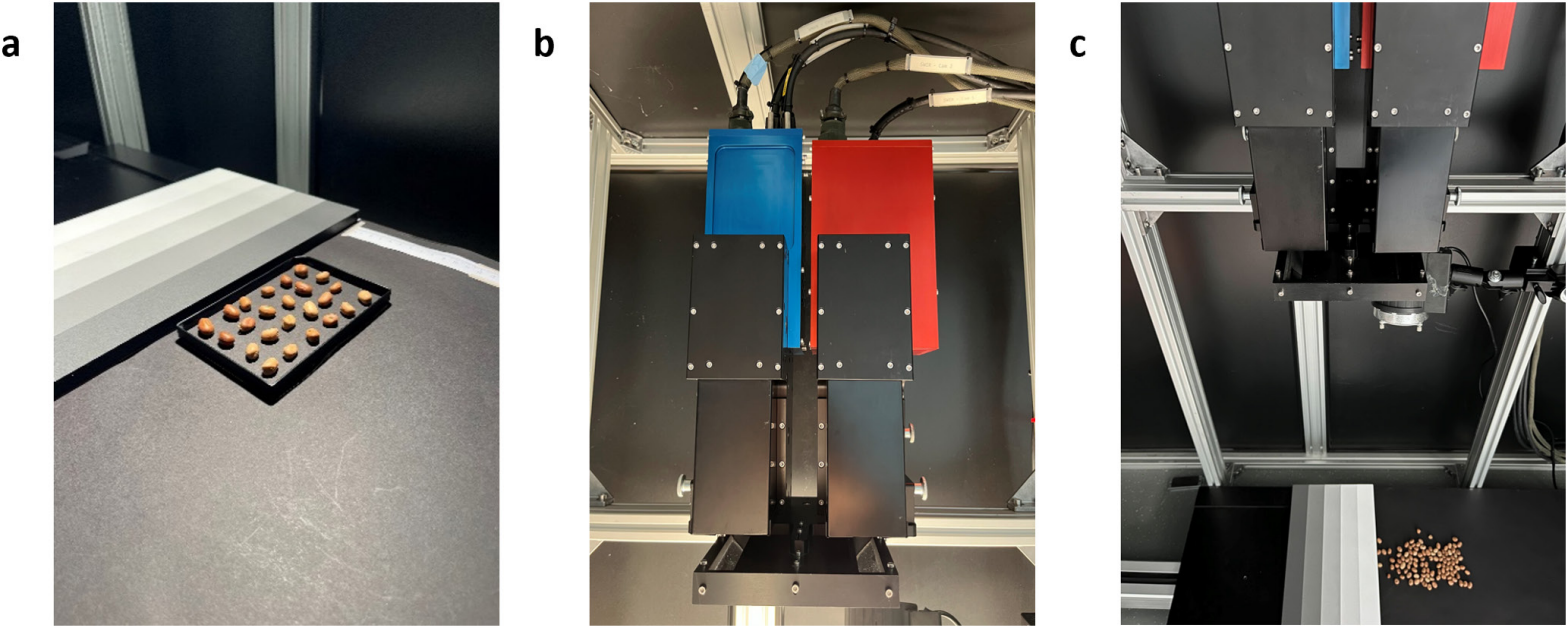
Setup for hyperspectral imaging. (a) Positioning of seeds. (b) Hyperspectral cameras. (c) Overview setup.

Each measurement was calibrated using the Zenith Polymer diffuser (white target), which has known reflectance, and the reflectance of the target was calculated using the following Equation (1)

(1) $$R_{\lambda }=\frac{I_{\lambda }-I_{\lambda }^{\mathrm{DC}}}{I_{\lambda }^{W}-I_{\lambda }^{\mathrm{DC}}}$$

Here, $I_{\lambda }$ is the image pixel intensity at wavelength λ, $I_{\lambda }^{\mathrm{DC}}$ is the so‐called “dark current”, i.e., the intensity measured with the shutter closed, and $I_{\lambda }^{W}$ is the intensity recorded from the white target. To remove the background, a segmentation scenario for the seed material was developed using the software perClass Mira (perClass BV, Delft, the Netherlands). For this purpose, a subset of images was manually annotated, and a reflectance‐based scenario was trained to classify each spectral pixel as either seed or background. For each seed, the mean reflectance, the reflectance standard deviation, and the seed size were extracted from the images using perClass Mira object feature selection.

### 2.4 Leaf Samples and DNA Extraction

Leaf samples from plants grown under field or greenhouse conditions were collected in 1.1 mL deep‐well tubes on dry ice, using 10 leaf discs with a diameter of 5 mm per sample. Samples were freeze‐dried for 2 days and extracted with the BioSprint 96 DNA Plant Kit from Qiagen (Düsseldorf, Germany) according to the manufacturer's instructions. The DNA concentration of five samples was measured utilizing the Qubit dsDNA BR Assay Kit from Invitrogen (Thermo Fisher Scientific, Waltham, MA, USA) as a reference, and a standard dilution to 40 ng/μL was performed.

### 2.5 Syn‐1 Classification

Based on SNP chip analysis of the parental lines conducted by SGS Fresenius Institute GmbH TraitGenetics Section (Gatersleben, Germany), Kompetitive Allele Specific PCR (KASP) markers were designed to distinguish between parental components of Syn‐1 combinations. For each Syn‐1 combination, one marker was developed that could classify its offspring as homozygous (corresponding to one of its parental components) or heterozygous (F_1_ hybrid of the respective parents). KASP marker design was performed according to Makhoul and Obermeier (2022), and analysis followed the PCR programme in Table S1. To confirm the functionality of the KASP markers, DNA samples from the respective homozygous parents were added to each KASP analysis as a control. Data were analyzed using the QuantStudio Real‐Time PCR software from Applied Biosystems by Thermo Fisher Scientific (Waltham, MA, USA). The complete dataset including spectral data and Syn‐1 classification can be found in Table S4.

### 2.6 Machine Learning

The following algorithms and R packages were utilized to distinguish the homozygous parental components from the heterozygous F_1_ seeds from the Syn‐1 combinations: k‐nearest neighbor (KNN) (r‐package “gmodels” (Warnes et al. 2024)), naive Bayes (NB) (r‐package “e1071” (Meyer et al. 2024)), decision tree with the algorithm C5.0 (DT) (r‐package “C50” (Kuhn and Quinlan 2025)), random forest (RF) (r‐package “randomForest” (Liaw and Wiener 2002)), and support vector machine (SVM) (r‐package “kernlab” (Karatzoglou et al. 2004)). We performed a fivefold cross‐validation, repeated 10 times with different fold assignments.

Machine learning algorithms were evaluated using the metrics precision (2), recall (3), F_1_ score (4), and accuracy (5), all calculated from the confusion matrix. The metrics were calculated using the following formulas:

(2) $$\text{Precision}=\frac{\text{true positive}}{\left(\text{true positive}+\text{false positive}\right)}$$

(3) $$\;\text{Recall}=\frac{\text{true positives}}{\left(\text{true positives}+\text{false negative}\right)}$$

(4) $$F_{1}=2*\frac{\text{precision}*\text{recall}}{\text{precision}+\text{recall}}$$

(5) $$\text{Accuracy}=\frac{\text{true positive}+\text{true negative}}{\text{true positive}+\text{true negative}+\text{false positive}+\text{false negative}}$$

For differentiation of parental components based on spectral patterns, scenario performance during training was optimized using accuracy as the target metric. In contrast, for differentiation of the whole Syn‐1, with a focus on F_1_ seeds, the F_1_ score was used as the optimization metric during training, as it provides a balanced measure of F_1_ hybrid seed identification and correct classification.

During preprocessing, spectral data were filtered to reduce noise using Savitzky–Golay filtering, implemented via the R package “prospectr” (Stevens and Ramirez‐Lopez 2025). Hyperparameters for Savitzky–Golay filtering (window size, polynomial order, and derivative order) of each algorithm were tuned individually using a grid search. The corresponding hyperparameters are described in Table S2. Hyperparameters of the algorithm were tuned individually utilizing Bayesian hyperparameter optimization (R package “ParBayesianOptimization” (Wilson 2022)). The corresponding hyperparameters can be found in Table S3.

In the two stages, different approaches were taken to manage unbalanced numbers of samples in each class. In scenario 1, for differentiation of parental components based on spectral patterns, the number of samples was balanced by reducing the sample count to that of a single parent. For differentiation between all components of Syn‐1 combinations, with a focus on identifying F_1_ seeds, adjustment of sample numbers by oversampling was necessary as the algorithms tended to overlook F_1_ seeds represented by only a small number of individuals. In scenario 1, the samples for each cross were duplicated in the training set to increase their representation. Due to the higher number of individuals in scenarios 2 and 3, a balanced dataset was used based on the number of parents for differentiation of parental components of Syn‐1 and on the number of F_1_ seeds for differentiation within the Syn‐1 combinations. Syn‐1 combination NPZ‐BP3Y2023‐060 consisted of 82 F_1_ seeds, whereas Syn‐1 combination NPZ‐BP3Y2023‐007 consisted of 70 F_1_ seeds.

In scenario 1, the different Syn‐1 combinations were coded as dummy variables, constructed with the R package psych (Revelle 2025). This is described as parental information (P). In all scenarios, we tested the influence of seed weight and seed size data, analyzed and labeled together as (W). Seed size was approximated by multiplying the seed length by the seed width. In combination with spectral data, we tested scenario 1 with four datasets consisting of (1) spectral, weight, size and parental information (SWP), (2) spectral, seed weight and seed size (SW), (3) spectral and parental information (SP), and (4) only spectral data (S). In scenarios 2 and 3, in which only one Syn‐1 combination per scenario was used, only two datasets were necessary, consisting of (2) spectral, weight and size data (SW) and (4) only spectral data (S), as parental information would have been the same for each entry.

In Stage 1, the 18 Syn‐1 combinations were divided into two groups based on differences between the spectral data of their respective parental components, in order to investigate the influence of spectral differences between seeds of the respective parental components on the performance of the algorithms. The mean Euclidean distance of the spectral data was calculated between parental component 1 and parental component 2 of each respective Syn‐1 combination. The Syn‐1 combinations were ranked from most distant to least distant and split into two equal‐sized groups representing “distant” and “near” Syn‐1 combinations (Table 2). The algorithms were run for each group separately and tuned individually (Table S3).

**TABLE 2:** Assignment of Syn‐1 combinations into “distant” and “near” Syn‐1 combinations based on the mean Euclidean distance of the spectra of their respective parental components.

Distant	Near
NPZ‐BP3Y2023‐056	NPZ‐BP3Y2023‐107
NPZ‐BP3Y2023‐090	NPZ‐BP3Y2023‐113
NPZ‐BP3Y2023‐093	NPZ‐BP3Y2023‐054
NPZ‐BP3Y2023‐007	NPZ‐BP3Y2023‐001
NPZ‐BP3Y2023‐053	NPZ‐BP3Y2023‐023
NPZ‐BP3Y2023‐006	NPZ‐BP3Y2023‐020
NPZ‐BP3Y2023‐065	NPZ‐BP3Y2023‐021
NPZ‐BP3Y2023‐047	NPZ‐BP3Y2023‐116
NPZ‐BP3Y2023‐060	NPZ‐BP3Y2023‐036

### 2.7 Principal Component Analysis

To visualize the spectral data, PCA was performed on scenario 1 data for all Syn‐1 combinations using modified Rogers' genetic distance (Goodman and Stuber 1983; Wright 1978). For scenarios 2 and 3, additional PCAs were performed including all samples of the respective Syn‐1 combinations.

## 3 Results

### 3.1 Visualization of Spectral Data

Visualization of Syn‐1 combinations by PCA revealed distinct Syn‐1 combination‐specific differences. When the spectra from parental lines formed two separate PCA clusters, spectra from the corresponding F_1_ seeds were generally positioned within these clusters, indicating a strong maternal effect on the spectral signature of the seeds (which also encompasses the seed color). Some Syn‐1 combinations, however, exhibited unclear cluster separation or overlapping clusters (Figure S3). Maternal effects were further evident in line graphs showing the mean spectra of the parental components and their F_1_ seeds for each Syn‐1 combination. The parental lines formed two distinct spectral profiles, with the spectra of F_1_ seeds typically lying between the mean spectra of the two parental components of each Syn‐1 combination. Occasionally, F_1_ seeds exhibited higher or lower excitation at specific wavelengths, suggesting the presence of F_1_ seed–specific spectral characteristics (Figure S4).

### 3.2 Stage 1

#### 3.2.1 Data Structure

In our dataset, the percentage of F_1_ seeds among the Syn‐1 combinations ranged from 13.5% to 26.9%, with a mean of 17.9% among the F_1_ seeds analyzed. The proportion of homozygous (self‐pollinated) seeds among the parental components ranged from 11.8% to 66.7%, with a mean of 41% per parental component per Syn‐1 combination (Table 3).

**TABLE 3:** Data structure of analyzed Syn‐1 combinations with the percentage of F_1_ seeds, parental component 1 and parental component 2 within each combination and tested *p* values from a *t*‐test.

Syn‐1 combination	F_1_ (%)	P1 (%)	P2 (%)	*p* values weight			*p* values volume		
				F_1_ ‐ P1	F_1_ ‐ P2	P1 ‐ P2	F_1_ ‐ P1	F_1_ ‐ P2	P1 ‐ P2
NPZ‐BP3Y2023‐001	14.6	62.5	22.9	0.7247	0.0095	0.0006	0.8825	0.0006	< 0.0001
NPZ‐BP3Y2023‐006	12.5	40.6	46.9	0.6035	0.8474	0.5839	0.0735	< 0.0001	0.3866
NPZ‐BP3Y2023‐007	26.9	47.3	25.8	0.6452	0.3769	0.5794	0.0758	0.4246	0.0027
NPZ‐BP3Y2023‐020	13.5	55.2	31.3	0.1095	0.9019	0.0246	0.0194	0.7476	0.0001
NPZ‐BP3Y2023‐021	15.8	52.6	31.6	0.0516	0.2202	0.3456	0.1430	0.7810	0.0998
NPZ‐BP3Y2023‐023	21.9	35.4	42.7	0.0851	0.9191	0.0345	0.0595	0.6014	0.0718
NPZ‐BP3Y2023‐036	24.5	45.7	29.8	0.4735	0.6174	0.1390	0.7408	0.4120	0.5230
NPZ‐BP3Y2023‐047	14.7	44.2	41.1	0.0424	0.0076	< 0.0001	0.0274	< 0.0001	0.0007
NPZ‐BP3Y2023‐053	13.5	27.1	59.4	0.5512	0.5883	0.8903	0.9539	0.2587	0.0759
NPZ‐BP3Y2023‐054	14.6	49.0	36.5	0.1179	0.1019	0.8637	0.9539	0.3093	0.1358
NPZ‐BP3Y2023‐056	14.9	33.0	52.1	0.1034	0.0128	< 0.0001	0.0002	0.0001	< 0.0001
NPZ‐BP3Y2023‐060	24.2	23.2	52.6	0.8321	0.3917	0.4570	0.5252	0.1688	0.4118
NPZ‐BP3Y2023‐065	14.9	39.4	45.7	0.7637	0.3019	0.0431	0.2402	0.9777	0.0780
NPZ‐BP3Y2023‐090	19.5	24.7	55.8	0.2128	0.3286	0.0031	0.3020	0.1559	0.0013
NPZ‐BP3Y2023‐093	21.5	47.3	31.2	0.0430	0.9274	0.0149	0.1072	0.9569	0.0495
NPZ‐BP3Y2023‐107	18.9	37.9	43.2	0.0570	0.6978	0.0018	0.0659	0.4810	0.0006
NPZ‐BP3Y2023‐113	21.5	66.7	11.8	0.7392	< 0.0001	< 0.0001	0.8674	< 0.0001	< 0.0001
NPZ‐BP3Y2023‐116	14.8	45.7	39.5	0.1794	0.2671	0.0002	0.3408	0.0903	0.0001

In some Syn‐1 combinations, we observed differences in seed weight and volume from the parental components (Table 3). Only cultivar NPZ‐BP3Y2023‐056 showed significant differences in seed volume for every comparison between F_1_ seeds and parental components and between the parental components. Otherwise, significant differences were generally observed between parental components but not between F_1_ seeds and their respective parents.

#### 3.2.2 Differentiation of Parental Seeds Based on Spectral Patterns

The accuracies of the differentiation of parental seeds based on spectral patterns varied for scenario 1, depending on the dataset and algorithm. The best‐performing algorithm was SVM with a mean accuracy of 66.8%. The best‐performing dataset was dataset 1 (SWP), including spectral, weight, volume, and parental information, with a mean accuracy of 69.9%. The weight and volume data did not influence the result significantly. The best‐performing combination across algorithms and datasets was RF with dataset 1, with a mean accuracy of 90.5%, ranging from 85.4% to 95.1% (Figure 2).

**FIGURE 2:**
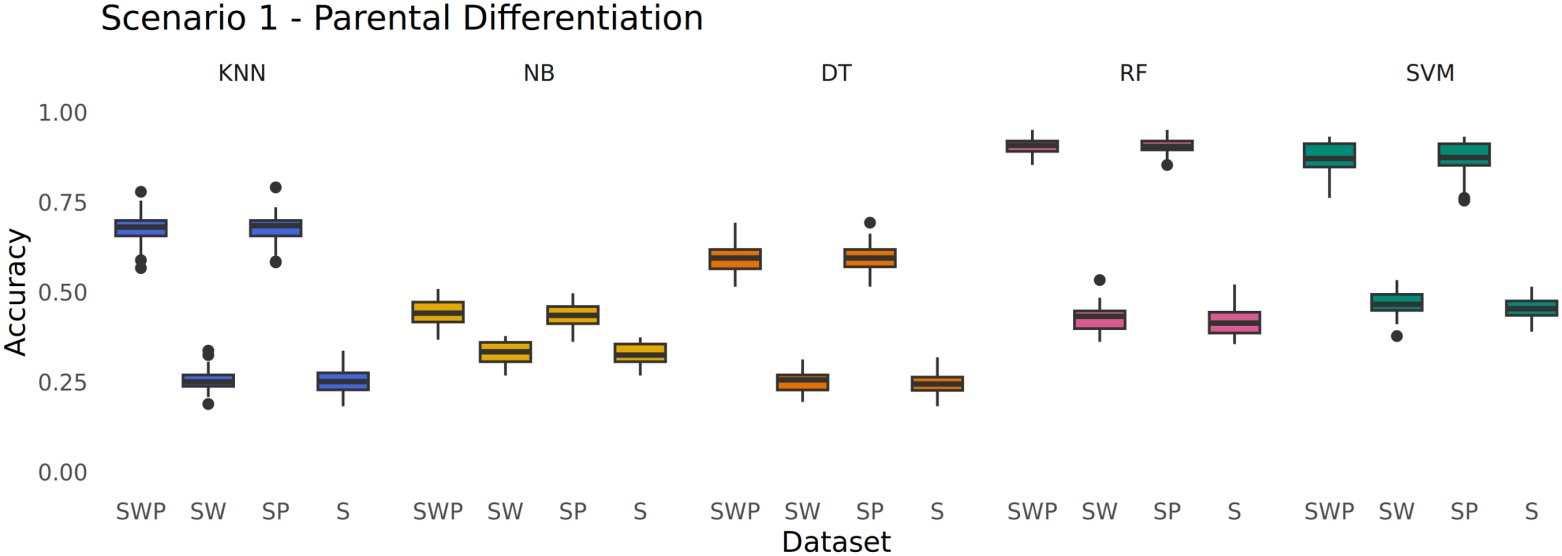
Parental differentiation of scenario 1. Comparison of prediction accuracy of differentiation of parental seeds of Syn‐1 combinations across the different datasets including spectral, weight, volume and parental information (SWP), spectral, weight and volume (SW), spectral and parental information (SP), and only the spectra (S) and different algorithms KNN (blue), NB (yellow), DT (orange), RF (pink), and SVM (green) for scenario 1.

#### 3.2.3 Differentiation of Syn‐1 Seeds Based on Spectral Patterns

Including all Syn‐1 seeds (corresponding to F_1_ seeds and self‐pollinated parental components) in the classification tasks reduced the predictability (Figure 3). The F_1_ score combines precision, reflecting how many of the predicted F_1_ seeds were true F_1_ hybrids, and recall, which indicates how many of the actual F_1_ seeds were correctly identified. Overall, the ability to distinguish self‐pollinated parental seeds from F_1_ hybrid seeds was poor, as the highest F_1_ score was only 19.7%, achieved by the SVM with dataset 1 (SWP). The best mean F_1_ score across datasets (12.7%) was obtained via SVM, while the best dataset was dataset 3 (SP). Inclusion of seed weight and volume did not significantly influence the prediction accuracies (Figure 3a). The most accurate prediction algorithm was SVM with 61.0% accuracy, while the most accurate predictions were achieved in dataset 1 (SWP) with 67.0%. The best combination was RF with dataset 1 (SWP) with 78.8% accuracy (Figure 3c). Including weight and volume in the datasets did not significantly influence the predictions.

**FIGURE 3:**
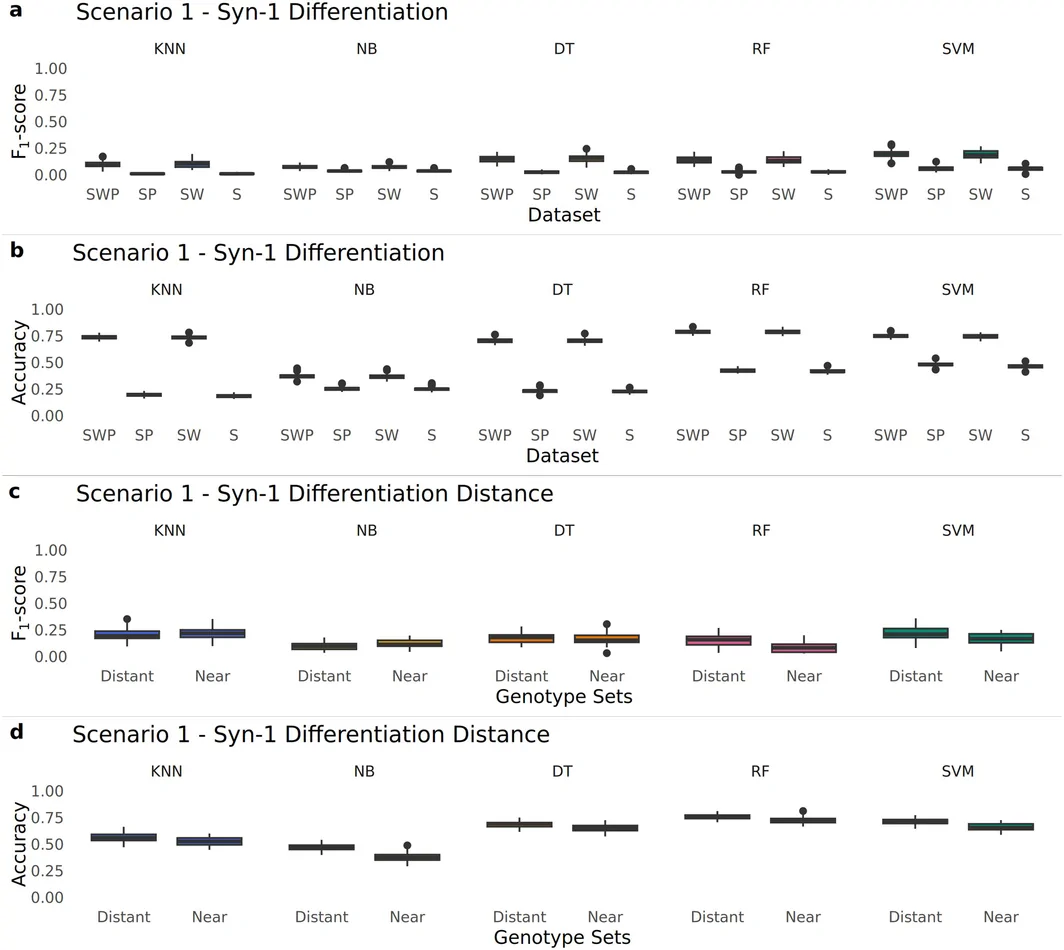
Syn‐1 differentiation of scenario 1. Comparison of (a) F_1_ score and (b) prediction accuracy of homozygous/heterozygous components of Syn‐1 combinations across the different datasets, including spectral, weight, volume, and parental data (SWP, SW, SP, and S). (c) F_1_ score and (d) prediction accuracy for scenario 1 separated into two types of Syn‐1 combinations based on PCA in “distant” or “near” Syn‐1 combinations. All predictions were run utilizing different algorithms: KNN (blue), NB (yellow), DT (orange), RF (pink), and SVM (green) for scenario 1.

The Syn‐1 combinations were separated into “distant” or “near” groups based on the Euclidean genetic distance between their parental components, differences that were already visible in their respective PCAs (Figure S3). Superior F_1_ scores were observed for “distant” groups with the algorithms DT, RF, and SVM. The best‐performing algorithm in this respect was KNN with 21.1%, while the best combination was KNN with “near” genotypes at 21.8% F_1_ score (Figure 3b). “Distant” groups of Syn‐1 combinations consistently showed better accuracies with a mean of 63.8% compared to “near” Syn‐1 combinations at 58.7%. Highest accuracy (75.5%) was achieved for predicting “distant” Syn‐1 combinations with the RF algorithm (Figure 3d).

### 3.3 Stage 2

#### 3.3.1 Data Structure

In this stage, we analyzed the Syn‐1 combinations NPZ‐BP3Y2023‐060 and NPZ‐BP3Y2023‐007 with a higher number of samples per Syn‐1 combination. NPZ‐BP3Y2023‐060 was represented by a total of 293 seeds, showing an outcrossing rate of 14.72%, while NPZ‐BP3Y2023‐007 was represented by 299 seeds and an outcrossing rate of 19.8%.

#### 3.3.2 Differentiation of Parental Seeds Based on Spectral Patterns

Differentiation between the parental components based on spectral patterns improved in scenario 2 in comparison to scenario 1. The best‐performing algorithm was SVM with a mean accuracy of 95.6%. Dataset 4 (S) performed slightly better with 92.7% accuracy in comparison to dataset 2 (SW) with 92.6%. The best‐performing combination across algorithms and datasets was SVM with dataset 2 (SW), with a mean accuracy of 95.6% ranging from 88.5% to 100.0% (Figure 4a). Accuracies of differentiation between parental components based on spectral patterns improved further in scenario 3. The best‐performing algorithm was RF with a mean accuracy of 98.9%. Datasets 2 (SW) and 4 (S) performed equally well with 98.6% accuracy. The best‐performing combination across algorithms and datasets was RF with datasets 2 (SW) and 4 (S), with a mean accuracy of 98.9% ranging from 95.4% to 100.0% (Figure 4b).

**FIGURE 4:**
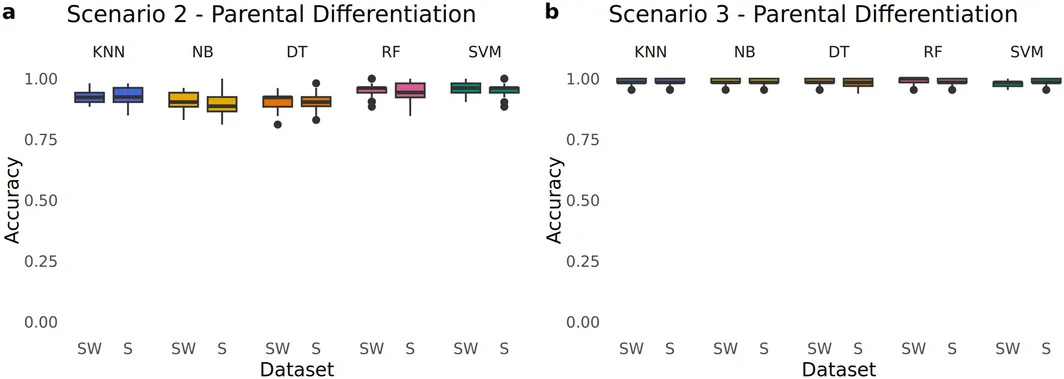
Parental differentiation of scenarios 2 and 3. Comparison of accuracy of differentiation of parental components of Syn‐1 combinations using (a) scenario 2 and (b) scenario 3 across the different datasets including spectral, weight and volume data (SW and S) and different algorithms: KNN (blue), NB (yellow), DT (orange), RF (pink), and SVM (green).

#### 3.3.3 Differentiation of Syn‐1 Seeds Based on Spectral Patterns

The accuracy of the differentiation between F_1_ seeds and their parental components based on the spectral patterns was lower than for comparisons between parental components but was improved in scenarios 2 and 3 in comparison to scenario 1. For scenario 2 with the Syn‐1 combination NPZ‐BP3Y2023‐060, the F_1_ score was improved to 38.6% with RF. Dataset 2 (SW) had the highest mean F_1_ score, at 33.5%. The best combination was SVM with dataset 2 (SW), at 38.7% F_1_ score (Figure 5a). For scenario 3, the Syn‐1 combination NPZ‐BP3Y2023‐007, the mean F_1_ score was highest for KNN, at 36.8%; datasets 2 (SW) and 4 (S) shared the highest mean F_1_ score, at 29.2%, while dataset 4 (S) achieved a higher maximum F_1_ score of 60.9%. The best combination was DT with dataset 2 (SW), at 37.9% F_1_ score (Figure 5b). For scenario 2, NPZ‐BP3Y2023‐060, the highest accuracy was 61.8% with RF and dataset 4 (S), at 52.1%. The best combination with the highest accuracy was RF with datasets 2 (SW) and 4 (S), at 61.9% (Figure 5c). For NPZ‐BP3Y2023‐007 in scenario 3, the best algorithm was RF at 60.4%, and the best dataset was dataset 2 (SW) at 54.7% accuracy. The best combination of algorithm and dataset was RF with dataset 2 (SW), at 62.8% (Figure 5d).

**FIGURE 5:**
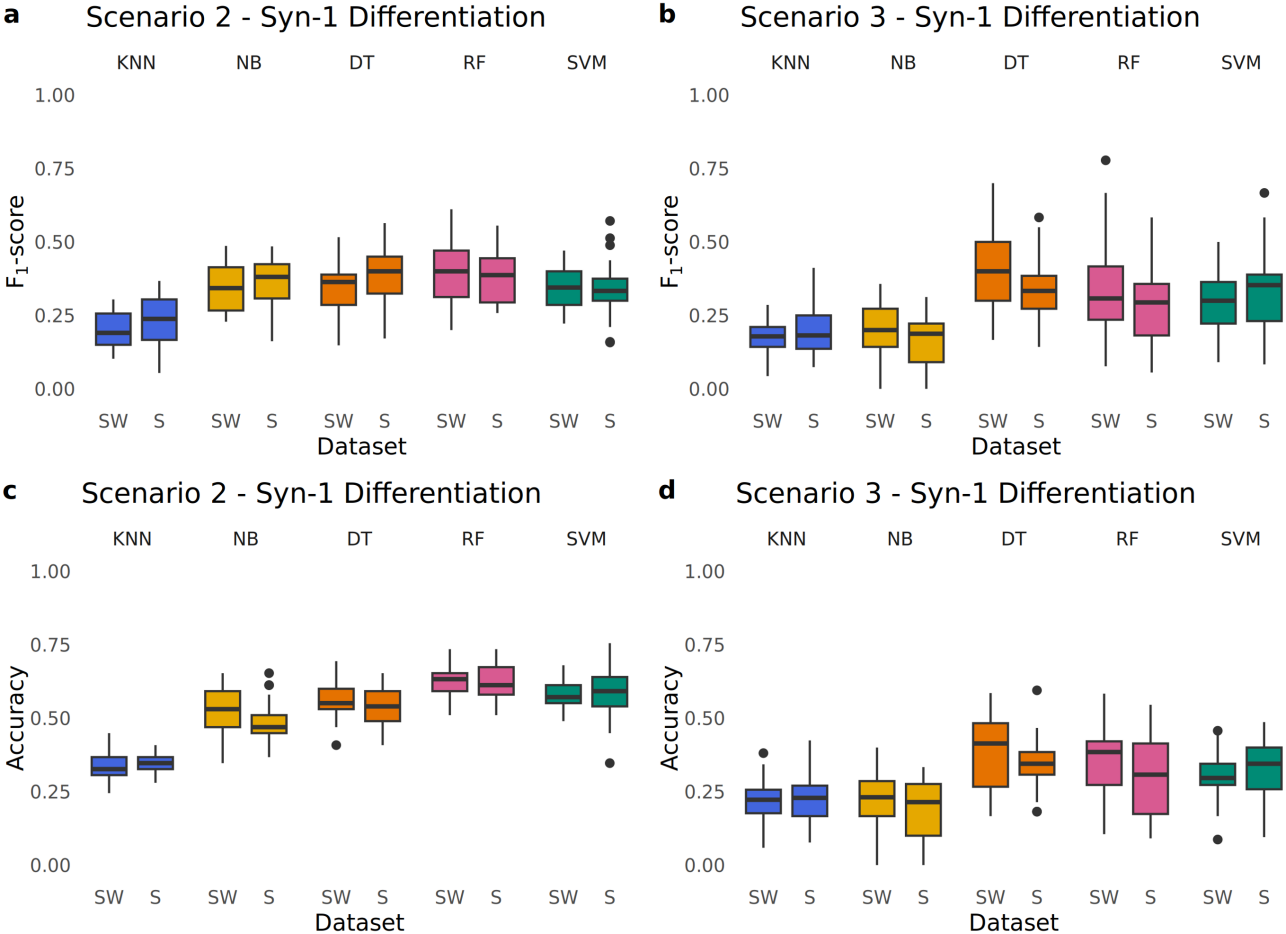
Syn‐1 differentiation of scenarios 2 and 3. Boxplots of prediction of F_1_ score for scenario 2 (a) and scenario 3 (b) and accuracy for scenario 2 (c) and scenario 3 (d) of differentiation of Syn‐1 seeds across the different datasets including spectral, weight, and volume (SW and S) and different algorithms KNN (blue), NB (yellow), DT (orange), RF (pink), and SVM (green).

To improve the F_1_ score, mean spectra were investigated to identify wavelengths showing F_1_ hybrid‐specific spectral patterns (Figure 6a,b). For scenario 2, RF performed best in this context with a 36.6% F_1_ score, and dataset 2 (SW) with 30.0%. The best combination for NPZ‐BP3Y2023‐060 (scenario 2) was RF with dataset 2 (SW), achieving an F_1_ score of 36.8% (Figure 6c). For scenario 3, genotype NPZ‐BP3Y2023‐007, the best algorithm was SVM at 39.6%, and the best dataset was dataset 2 (SW) at 37.8% F_1_ score. The best combination of algorithm and dataset was SVM with dataset 2 (SW), with an F_1_ score of 40.7% (Figure 6d). The accuracy of scenario 2 was 58.5% with the algorithm RF, while dataset 2 (SW) showed the highest accuracy for the datasets with SVM (47.5%). The best combination was DT with dataset 2 (SW) (accuracy 50.4%) (Figure 6e). In scenario 3, the highest accuracy among algorithms was 61.0% with RF, and between datasets, dataset 2 (SW) achieved a mean accuracy of 59.5%. The best combination was KNN with dataset 2 (SW), at 61.7% (Figure 6f).

**FIGURE 6:**
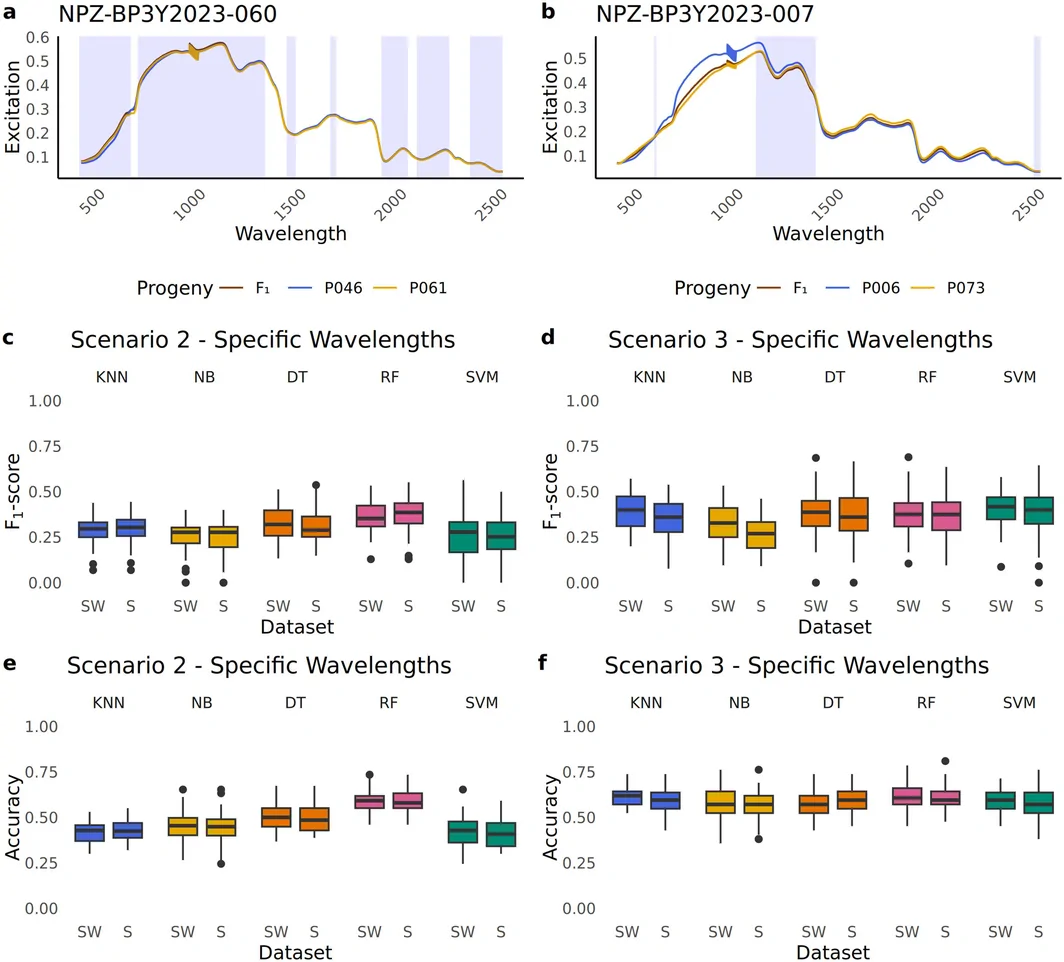
Wavelength‐specific Syn‐1 differentiation of scenarios 2 and 3. Line graph showing the mean spectra for (a) NPZ‐BP3Y2023‐060 and (b) NPZ‐BP3Y2023‐007 generated from the raw spectra, blue background indicating the wavelengths used for the prediction, brown lines for F_1_ seeds and blue and yellow for their parental lines. Boxplots of prediction of F_1_ score for scenario 2 (c) and scenario 3 (d) and accuracy for scenario 2 (e) and scenario 3 (f) of differentiation of Syn‐1 combinations across the different datasets including spectral, weight, and volume (SW and S) and different algorithms KNN (blue), NB (yellow), DT (orange), RF (pink), and SVM (green).

As the parental components of the Syn‐1 combination NPZ‐BP3Y2023‐007 could be separated based on PCA (Figure 7e), F_1_ seeds were assigned to one of the parental components, as it is hypothesized that maternal effects led to this clustering. This could not be validated as we only had a mixture of synthetic combinations rather than the exact progeny, which would have indicated the maternal and paternal parents. The parental component P006 of NPZ‐BP3Y2023‐007 had an approximate outcrossing rate of 13.29%. The best‐performing algorithm regarding F_1_ score was DT with 62.2% and dataset 4 (S) with 50.8%. The best combination was DT with dataset 2 (SW), at 62.2% F_1_ score (Figure 7a). The accuracy was highest with DT, at 64.6%, and with dataset 4 (S), at 53.9%. The best combination of algorithm and dataset was DT with dataset 4 (S), giving a prediction accuracy of 64.8% (Figure 7c). The parental component P073 had an approximate outcrossing rate of 27.61%. F_1_ score was highest with algorithm NB at 60.2%, and with dataset 2 (SW), at 43.4%. The best combination was NB with dataset 4 (S), giving an F_1_ score of 60.4% (Figure 7b). The prediction accuracy was also highest for NB, at 58.8% and for dataset 2 (SW), at 43.7%. The best combination with the highest accuracy was NB with dataset 4 (S), at 59.0% (Figure 7d).

**FIGURE 7:**
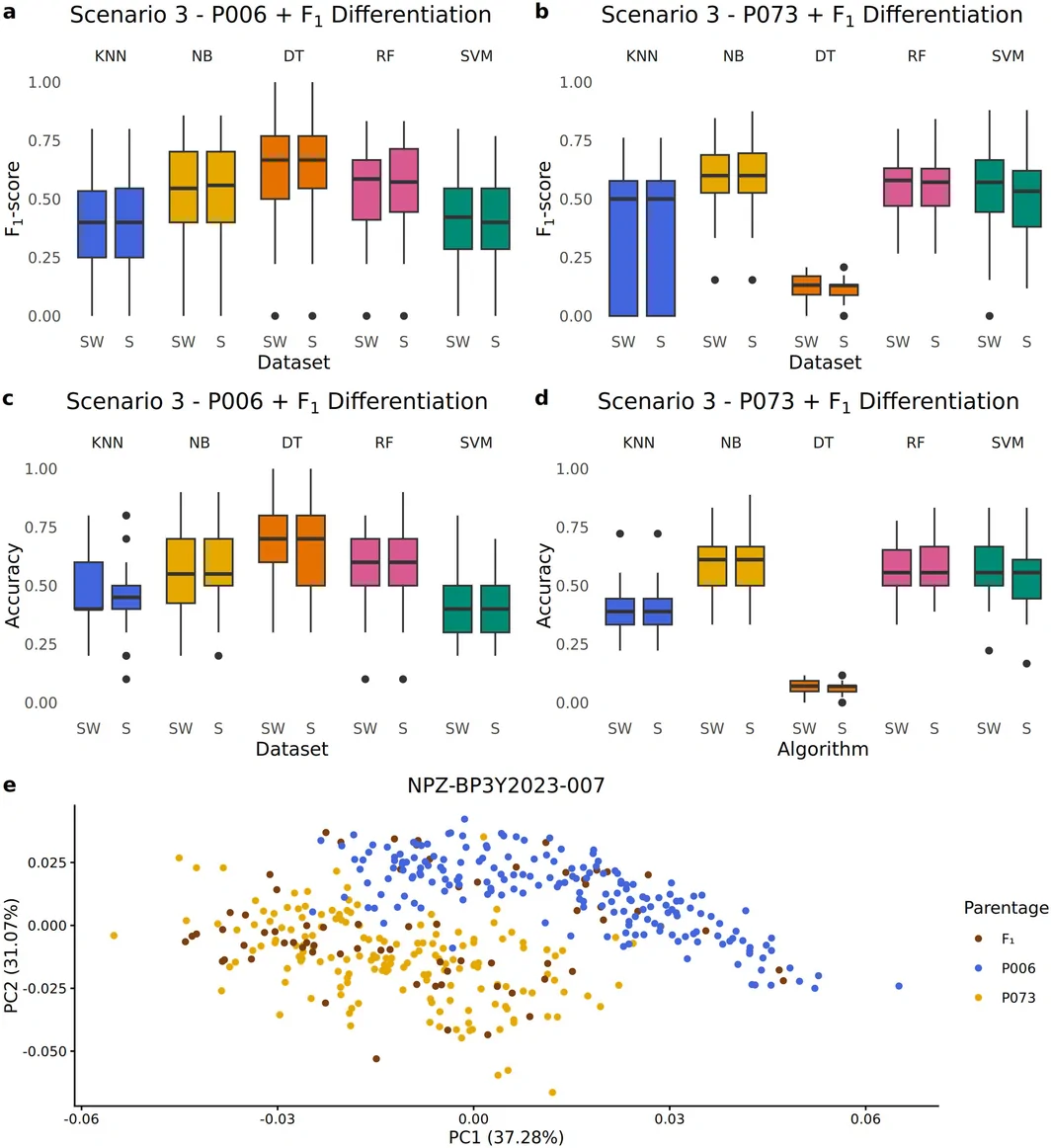
Parental‐specific Syn‐1 differentiation of scenario 3. (a–d) Boxplots describing the ability to differentiate F_1_ seeds from their parents using scenario 3, showing the F_1_ scores for (a) parental component P006 and (b) parental component P073, as well as the accuracy for (c) parental component P006 and (d) parental component P073 across different datasets including hyperspectral reflectance, weight, and volume (SW and S) and different algorithms KNN (blue), NB (yellow), DT (orange), RF (pink), and SVM (green). (e) PCA of hyperspectral reflectance patterns from seeds representing F_1_ seeds (brown points) and their respective parents (blue and yellow points, respectively) from the biparental Syn‐1 combination NPZ‐BP3Y2023‐007.

## 4 Discussion

### 4.1 Identification of Parental Components of Syn‐1 Combinations

Numerous studies have addressed the assessment of variety purity using spectral data. For instance, variety identification based on spectral analysis has been extensively documented in crops such as wheat, maize, okra, and loofah seeds (Zhao, Que, et al. 2022; Yang et al. 2015; Nie et al. 2019). These studies often integrate spectral and spatial features, achieving classification accuracies of up to 98%. In the present study, Stage 1 demonstrated that, despite the limited sample size per group, the parental components of faba bean Syn‐1 combinations could be classified with an accuracy of 90.5%. The scenarios developed in Stage 2 further enhanced predictive performance, reaching accuracies of up to 98.9%. Notably, while differences among Syn‐1 combinations were observed, prediction accuracies remained consistently high.

### 4.2 Are Xenia Effects in Faba Bean Revealed by Spectral Reflectance Patterns?

The xenia effect in faba bean seeds was described by Duc et al. (2001), who observed an influence of paternal pollen on volume and number of cotyledon cells for all analyzed faba bean genotypes. However, an impact on seed weight was limited to small‐seeded paternal components. In our study, no significant differences were detected between parental components and F_1_ seeds across the 18 Syn‐1 combinations examined, except for Syn‐1 combination NPZ‐BP3Y2023‐056 (Table 3). For this Syn‐1 combination, the volume differed significantly not only between parental components and F_1_ seeds but also between the parental components themselves. Heterosis for F_1_ seed weight, particularly when parents are genetically divergent, has been described by Dieckmann and Link (2010). While such positive effects on seed weight are documented in hybrid seeds, they were not reflected in the predictive performance of our scenarios. Neither seed weight nor volume exhibited a significant influence on our prediction performance. In different species such as maize, an increased seed weight in hybrid seed is also observed and is considered an early form of heterosis (Bulant and Gallais 1998).

A xenia effect on seed color, as observed in pea with interspecific crosses (Sari et al. 2023), has not yet been reported in faba bean, where seed color is determined by maternal inheritance (Ricciardi et al. 1985). This is consistent with soybean, in which seed color has also been described as maternally inherited (Kumar et al. 2025). These observations are supported by the clustering patterns in PCAs, where parental components formed two distinct clusters, and F_1_ seeds fell into one or the other of the parental clusters. Under these conditions, the expected outcome would be 33% correct classifications through random assignment in Stage 2 of this study for the F_1_ score. However, our scenario achieved a mean F_1_ score of 39%, indicating that a xenia effect may be present but either minimal or not detectable within the visible spectrum. This is further supported by the differentiation of Syn‐1 combinations with separated parental components, where random assignment would have had an accuracy or F_1_ score of 50%, yet our algorithms exceeded this threshold, reaching up to 64% accuracy. To improve predictive performance, a potential next step could involve the development of a hybrid convolutional neural network designed to capture both the spectral reflectance and the structural or textural features of seeds. Zhao, Que, et al. (2022) successfully applied this approach to wheat variety identification, demonstrating that a model combining spectral and spatial features outperformed those based on either feature set alone.

### 4.3 Limitations of the Study

This study aimed to identify whether differentiation between the components of Syn‐1 combinations was possible. It showed moderate prediction performance for identification of F_1_ seeds and high performance for differentiation between parental components alone within the Syn‐1 combinations. The study focused on only one preprocessing method and a few machine learning algorithms; therefore, the prediction performance could probably be improved through some of the following approaches. To improve the prediction performance, the sample size could be increased, as already observed: the two additional models in scenarios 2 and 3 showed increased prediction performance compared to scenario 1. Further traits, such as hilum color, surface texture, exact shape, or a more precise measurement of the size, could potentially improve the prediction further.

In this study, the Savitzky–Golay filter was applied, and the optimal combination of window size, polynomial order, and derivative was investigated individually for each approach. In addition to this method, however, several other preprocessing techniques for NIR data have also shown good performance. Other preprocessing methods, such as standard normal variate transformation, multiplicative scatter correction, and normalization, have been shown to yield good performance in seed classification across different harvest years. In that study, Savitzky–Golay filtering was also among the top‐performing preprocessing methods (Zhu et al. 2024).

To further improve such classifications, different machine learning models could be applied, such as neural networks, for example, convolutional neural networks (Zhao, Que, et al. 2022) or autoencoders (Kabiso and Ko 2025); partial least squares discriminant analysis (Kong et al. 2013; Zhu et al. 2019); linear discriminant analysis (de Medeiros et al. 2020); or various boosting methods, for example, XGBoost, LightGBM, and CatBoost (Wu et al. 2022), all of which have already been established for seed variety identification or seed quality classification. Due to their higher complexity, these methods could be expected to yield higher prediction accuracies for differentiation of F_1_ seeds, whereas the algorithms used in this study already gave good results for differentiation of parental components.

Another limitation of this study is the exclusive use of breeding material. Faba bean is a crop rich in diversity, which also includes seed color, ranging from violet, red, green, beige, brown, and black to spotted seed coats (Ricciardi et al. 1985). Furthermore, this variation extends to different seed sizes, even classifying germplasm into the small‐seeded Minor and large‐seeded Major groups (Link et al. 1996). This study did not investigate whether the generated results could be transferred to different germplasm or more diverse datasets; this needs to be done in future work to validate robustness and transferability of the presented approach. However, the studies by Duc et al. (2001) and Dieckmann and Link (2010) suggested that broader variation among analyzed individuals could lead to a more pronounced xenia effect and, hypothetically, to improved prediction performance, although this would require further investigation to confirm. Through all of these methods, there is potential to further improve the prediction performance, and thereby the method presented in this study, which could further increase faba bean yield as described below.

### 4.4 Faba Bean Breeding Applications

Faba bean is bred as synthetic cultivars, with a mean outcrossing rate of approximately 50% and a range from 7% to 82% (Link 1990; Link, Ederer, et al. 1994; Link, Stelling, and Ebmeyer 1994). An ability to distinguish F_1_ seeds from their homozygous parents could potentially be used to enrich F_1_ hybrid seeds in synthetic cultivars, thus potentially improving heterosis and seed yield (Link, Stelling, and Ebmeyer 1994). Moreover, the technique might also be useful as a selection method to identify parental components that show elevated outcrossing rates.

Considering the outcrossing rates of the Syn‐1 combinations used in this study, which ranged from 13.5% to 26.9%, an F_1_ score of 39%, as in scenario 2, would correspond to a hybrid proportion of 39% in the sorted cultivar. Despite the relatively modest prediction performance, this would still be expected to result in a yield increase, as previously described. Furthermore, such predictions or sorting could potentially reduce the number of propagation generations required for synthetic cultivars. Typically, Syn‐2 or Syn‐3 generations are sold to farmers (Stelling et al. 1994). If the desired yield level could be achieved as early as the Syn‐1 generation through seed sorting, this could save up to one generation of costly seed propagation.

Overall, while this method has not yet led to the implementation of a 100% pure hybrid cultivar, it could pave the way for hybrid‐enriched synthetic cultivars and demonstrate the potential of using hyperspectral images for seed sorting.

## 5 Conclusion

In conclusion, the differentiation of parental components in Syn‐1 combinations from biparental synthetic combinations via spectral patterns generally performs well. While the accuracy of the identification of F_1_ seeds is comparatively lower, it is nevertheless sufficient for potential enrichment of Syn‐1 seedlots with F_1_ hybrid seeds. This sorting approach could result in an increased proportion of F_1_ hybrid seeds within synthetic cultivars, potentially boosting yield due to heterosis. Better performance of Syn‐1 cultivars compared to Syn‐2 or Syn‐3 would reduce the number of propagation cycles required, saving time and costs in breeding programmers for synthetic cultivars. This study demonstrates the potential of seed hyperspectral reflectance data and machine learning to improve faba bean breeding, noting that different machine learning algorithms or preprocessing methods could potentially further improve prediction performance. Implementation in conjunction with automated seed sorting could already further streamline breeding processes and contribute to possible time, cost and yield advantages in the development of synthetic cultivars.

## Author Contributions

**Rica‐Hanna Schlichtermann:** conceptualization, methodology, formal analysis, investigation, writing – original draft. **Sebastian Warnemünde:** methodology, formal analysis, investigation. **Hanna Tietgen:** resources. **Gregor Welna:** resources. **Andreas Stahl:** supervision. **Benjamin Wittkop:** conceptualization, supervision. **Rod J. Snowdon:** conceptualization, supervision, funding acquisition. All authors revised the manuscript and approved the final manuscript.

## Funding

The work was funded by Grant 469336000 to R.J.S. from the Deutsche Forschungsgemeinschaft (DFG) for the International Research Training Group 2843 “Accelerating Crop Genetic Gain”.

## Disclosure

During the preparation of this work, the lead author used Le Chat from Mistral AI (Knowledge cutoff: November 2024) in order to improve language and readability. After using this tool, the authors thoroughly reviewed and edited the content as needed. The authors take full responsibility for the content of the published article.

## Conflicts of Interests

The authors declare no conflicts of interest.

## Supporting information




**Table S1:** PCR‐programme for KASP analysis.
**Table S2:** Tuned hyperparameters for each prediction and Savitzky–Golay filtering.
**Table S3:** Tuned hyperparameters for each prediction and algorithm.
**Table S4:** Raw data used for the predictions.

**Figure S1:** Example plates from the Syn‐1 combinations.
**Figure S2:** Parental genotypes used for the generation of Syn‐1 combinations.
**Figure S3:** Principal component analysis of hyperspectral reflectance patterns of Syn‐1 combinations.
**Figure S4:** Mean hyperspectral reflectance patterns of Syn‐1 combinations.

## Data Availability

The dataset generated during the current study is available in the Supporting Information.
