# Supplementary material for: Progeny Differentiation in Faba Bean Using Hyperspectral Images and Machine Learning
Source: Plant Direct. 2026 Oct 4;10(10):e70193. doi: 10.1002/pld3.70193 (PMC13634928; doi:10.1002/pld3.70193)
Supplement: Supplementary file 1 [file PLD3-10-e70193-s001.pdf]

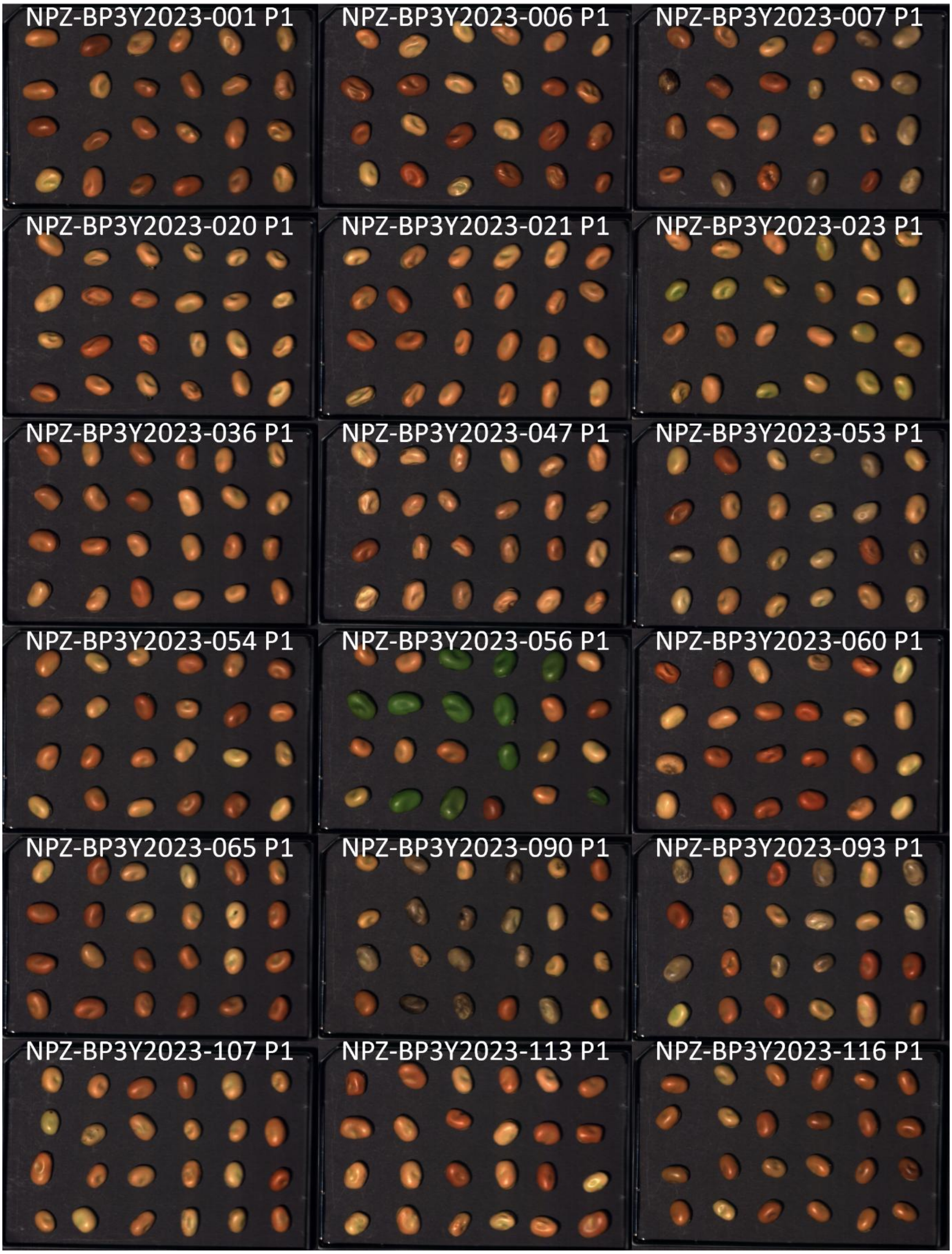

Figure S1: Example plates from the Syn-1 combinations. The first plate of each Syn-1 mixture is shown. Seeds are numbered from 1 to 24, starting in the upper left corner and proceeding row by row from left to right, ending at position 24 in the lower right corner.

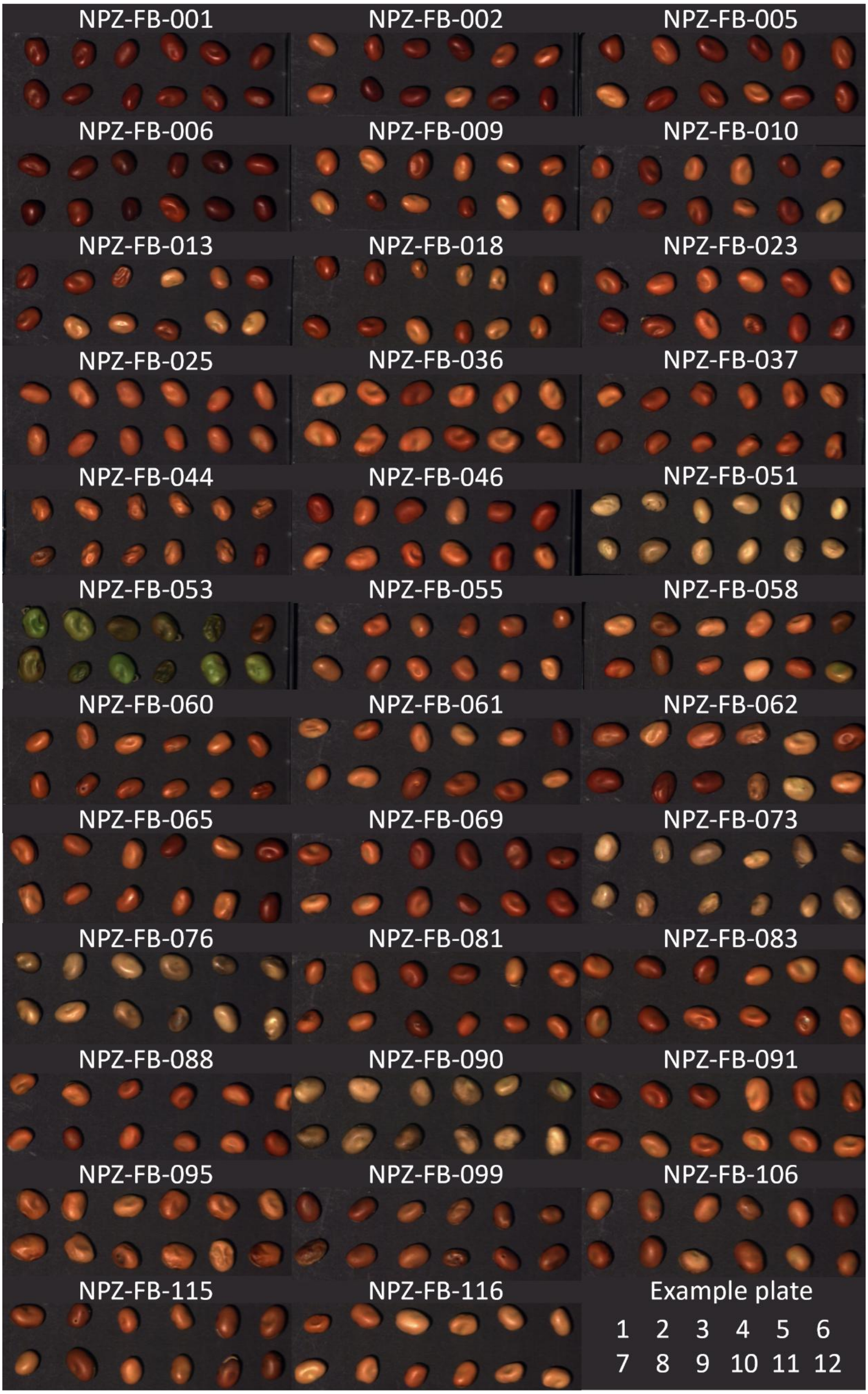

Figure S2: Parental genotypes used for the generation of Syn-1 combinations. Shown are the parental genotypes and an example plate demonstrating how individual seeds were numbered.

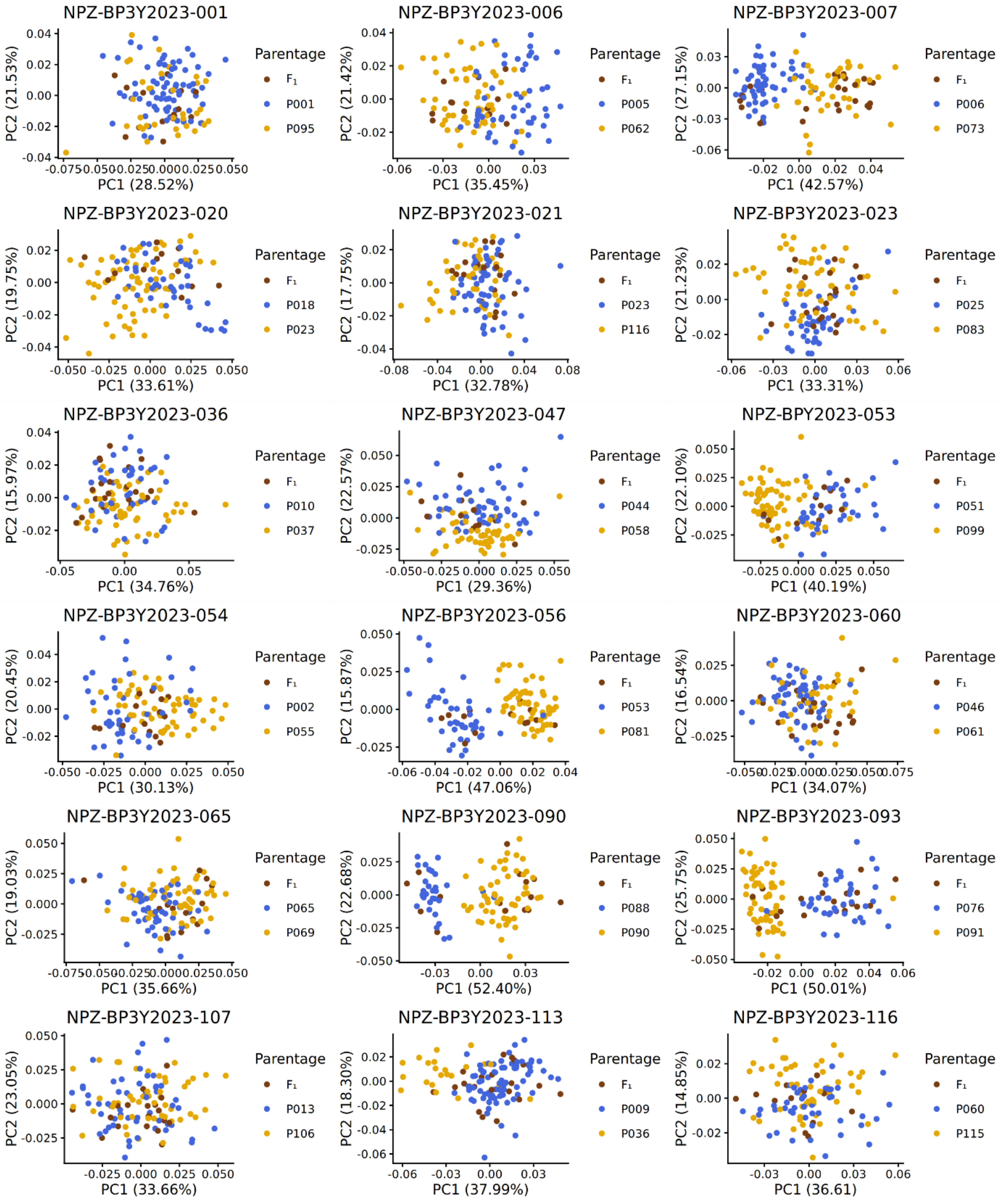

Figure S3: Principal component analysis of hyperspectral reflectance patterns of Syn-1 combinations. PCA of hyperspectral reflectance patterns from seeds representing F_1 hybrids (brown points) and their respective parents (blue and yellow points, respectively) from 18 biparental Syn-1 synthetic faba bean combinations.

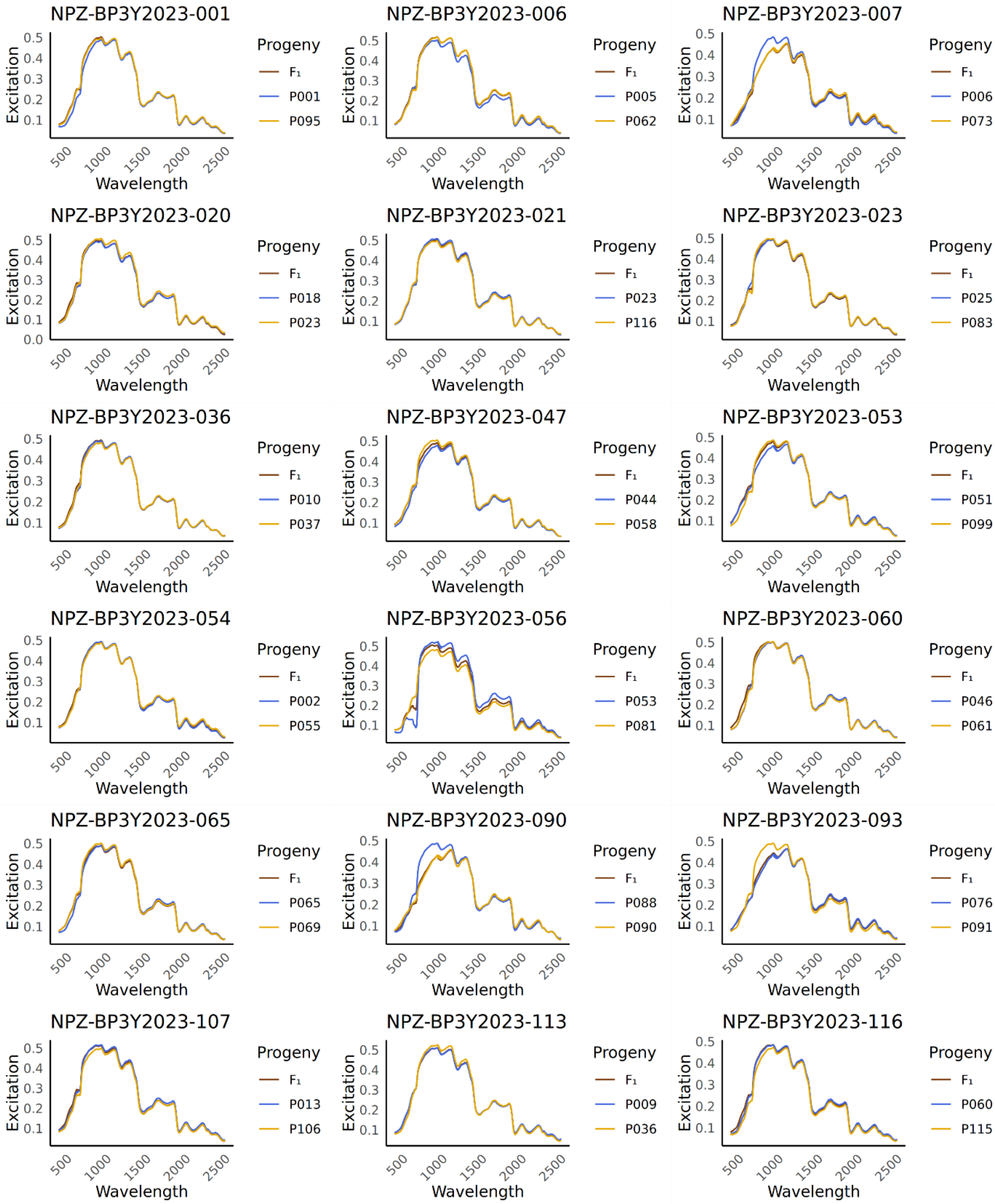

Figure S4: Mean hyperspectral reflectance patterns of Syn-1 combinations. Mean hyperspectral reflectance patterns from seeds representing F_1 hybrids (brown lines) and their respective parents (blue and yellow lines, respectively) from 18 biparental Syn-1 synthetic faba bean combinations based on the raw spectra.
